# Leveraging the Age-Inclusivity Domains of Higher Education Model for Personnel and Community Engagement

**DOI:** 10.1093/geroni/igaf122.1269

**Published:** 2025-12-31

**Authors:** Katherina Nikzad-Terhune, Allyson Graf, Joann Montepare

## Abstract

At a time of contraction, return to work orders, and shrinking budgets, universities must be innovative in supporting age-diverse employees and sustaining employee morale. The age-inclusivity domains of higher education (AIDHE) model provides a framework for how institutions of higher education may consider age-inclusive practices across seven institutional domains, including personnel and community engagement. This symposium highlights ways in which these two components of the AIDHE model can help institutions craft a university work environment that is responsive to the needs of an age-diverse workforce. Nikzad-Terhune and colleagues (Northern Kentucky University) evaluated caregiving needs in a campus-wide assessment and focus here on data-driven recommendations on how universities can support family caregiving beyond FMLA. A study by Swanson and June (Central Connecticut State University) furthers the work of the community engagement domain at their Age-Friendly University by exploring the utilization and experience of those aged 62+ years who have registered for a course through the tuition waiver program in the past three years. Graf and colleagues (Northern Kentucky University) will apply the AIDHE model to results from a survey of mid-career faculty to illustrate how institutions can apply an age-inclusive lens to employee retention, development, and support. Kaskie and colleagues (University of Iowa) will present data on age-inclusive management strategies (the AIMS project), which seeks to educate employers about the aging workforce and support the adoption of management strategies targeting older employees. Joann Montepare (Lasell University), Chair of the GSA Age Inclusivity in Higher Education Workgroup, will be the discussant. Age Inclusivity in Higher Education Interest Group Sponsored Symposium

